# Concentrations of TSP-Bound Metals in Four Urban Residential Locations in Seoul, Korea

**DOI:** 10.1100/tsw.2010.70

**Published:** 2010-05-04

**Authors:** Hang Thi Nguyen, Chan Goo Park, Jin-A Kim, Je-Seung Lee, Jin-Hong Lee, Ki-Hyun Kim

**Affiliations:** ^1^ Department of Earth and Environmental Sciences, Sejong University, Seoul, Republic of Korea; ^2^ Seoul Metropolitan Institute of Public Health and Environment, Chungnam National University, Daejeon, Republic of Korea; ^3^ Department of Environmental Engineering, Chungnam National University, Daejeon, Republic of Korea

**Keywords:** metal, particulate matter, residential area, traffic, Seoul

## Abstract

Concentrations of 17 trace metals bound in total suspended particulate (TSP) were measured at four urban residential locations (Jong Ro [JR], Gwang Jin [GJ], Gang Seo [GS], and Yang Jae [YJ]) in Seoul, Korea from February to July 2009. The maximum concentrations of metals were recorded by Fe in the range of 2599 (JR) to 2914 ng m^-3^ (GJ), while the least values were observed from Ag or Co with a few ng m^-3^. The relative ordering of the mean concentration (ng m^-3^) at these sites is generally found on the order of Fe > Zn > Ba > Mn > Pb > Cu > B > Cr > Ni > Sr >V > As > Li > Cd > Mo > Co > Ag or with a few exceptions (e.g., a reversal between Ba and Mn or between Ni and Sr). Calculation of the enrichment factor suggests the significant role of man-made processes on such metals as Cd, Zn, and Pb. Inspection of the temporal patterns indicates the peak occurrence of most metals during the spring season due in part to the Asian Dust (AD) event. However, according to the factor analysis, sources of these metals were dominated by both resuspended soil/road dust and the combustion of fossil fuels. The overall results of our study suggest that the interaction between the environmental conditions and roadside traffic activities are paramount in explaining the metal pollution in these urban residential areas.

## INTRODUCTION

Aerosol is known as a suspension or resuspension of fine solid particles or liquid droplets in air. At sufficiently large sizes, particulate matter (PM) can scatter and absorb sunlight to cause a reduction in visibility and sunrise and sunset reddening[1,2,3] in rural areas, while the weak visibility is prevalent in urban areas due to differences in man-made activities[1]. Aerosols are known to participate in a number of important atmospheric processes with their direct and indirect effects on the Earth's radiative budget (via scattering) or on the surface ozone concentrations[4]. For instance, an increase in aerosol loadings can lead to a decrease in the ozone concentration[5].

It is known that most harmful metals are introduced mainly by man-made activities such as metal refining, waste incineration, fossil fuel combustion, vehicle exhausts, and industrial processes (e.g., [6]).

Those metals present in particulate form are dispersed ubiquitously throughout the atmosphere until their removal by a variety of cleaning processes, including dry deposition, as well as scavenging and washout by rain[6,7,8]. Although natural sources (e.g., volcanism, erosion, surface winds, forest fires, etc.) are as significant as the man-made counterparts, the magnitude of metal emission by the former is yet more difficult to quantify. Nonetheless, the resuspension of surface dust is identified as the dominant compartment of the natural metal budget on a global scale. For instance, it is expected to account for >50% of Cr, Mn, and V, and >20% of Cu, Mo, Ni, Pb, Sb, and Zn[9].

Rapid urbanization, high population density, and an increasing rate of energy consumption have caused elevated emissions of PM and their components in certain regions of the world[10,11]. The combination of vehicular emissions and dust resuspension is recognized as constituting one of the most significant man-made sources of metal species[12,13,14,15,16]. Although diverse anthropogenic activities were allocated as the sources of the fine-sized PM, the evolution of such source processes has been detected in many urban areas on the globe[11,17,18].

In this study, the fundamental characteristics of metal distribution were investigated from four different urban residential areas using the concentration data of 17 metals (Ag, As, B, Ba, Cd, Co, Cr, Cu, Fe, Li, Mn, Mo, Ni, Pb, Sr, V, and Zn) bound with the total suspended particulate (TSP). The concentrations of these metal species were simultaneously determined with the various particulate components during the months of February through July 2009. These measurement data were analyzed in order to provide insight into the factors affecting the environmental behavior of metal species, along with their relationship with the complicated source activities under diverse environmental conditions of the urban residential areas.

## MATERIALS AND METHODS

### Site Characteristics

Monitoring of 17 metals bound with TSP samples was carried out at four different urban residential areas representing Jong Ro (JR: 127$^o$ 00.18’, 37$^o$ 34.19’), Gwang Jin (GJ: 127$^o$ 05.44’, 37$^o$ 32.40’), Gang Seo (GS: 126$^o$ 50.18’, 37$^o$ 32.11’), and Yang Jae (YJ: 127$^o$ 01.55’, 37$^o$ 27.51’) in Seoul, Korea (Fig. 1). As the highly urbanized, metropolitan, capital city of Korea (a total area of 605.5 $km^2$), Seoul records one of the highest population densities in the world (a population of about 10.3 million) with about 3.5 million households[19]. The vehicular densities in Seoul are large with a sum of about 2.3 million. A total of about 18,100 industrial factories occupy about 12% of the city area in Seoul[19]. The land use type of all four sites investigated in this study (Fig. 1) can be represented as the residential type. A brief inspection of each site indicates that none of them are directly affected by any type of strong sources other than traffic activity from adjacent roads (e.g., GJ and GS sites). The field campaigns for the collection of daily PM samples were made from the four sites on three consecutive days per month from February to July 2009: (1) February 23–25, (2) March 9–11, (3) April 6–8, (4) May 11–13, (5) June 9–11, and (6) July 6–8. During these sampling periods, some major pollutants (e.g., $SO_2$, NO, $NO_2$, $NO_x$, CO, $O_3$, $PM_{10}$, $PM_{2.5}$, $CH_4$, $NCH_4$, and THC) and the relevant meteorological parameters (e.g., temperature [TEMP], humidity [HUM], wind speed [WS], wind direction [WD], ultraviolet [UV], and solar) were also monitored simultaneously on a routine basis.

### Sampling and Analysis

The collection of TSP samples was made on a daily basis using a high-volume air sampler (SIBATA, HV-1000F, Japan) with the Quartz-type PALLFLEX membrane filter. The collection of each TSP sample was initiated from 1500 h at a flow rate of 800 L $min^{-1}$ for a 24-h interval. For each month, TSP samples for the analysis of metal species were collected for each day over three continuous days.

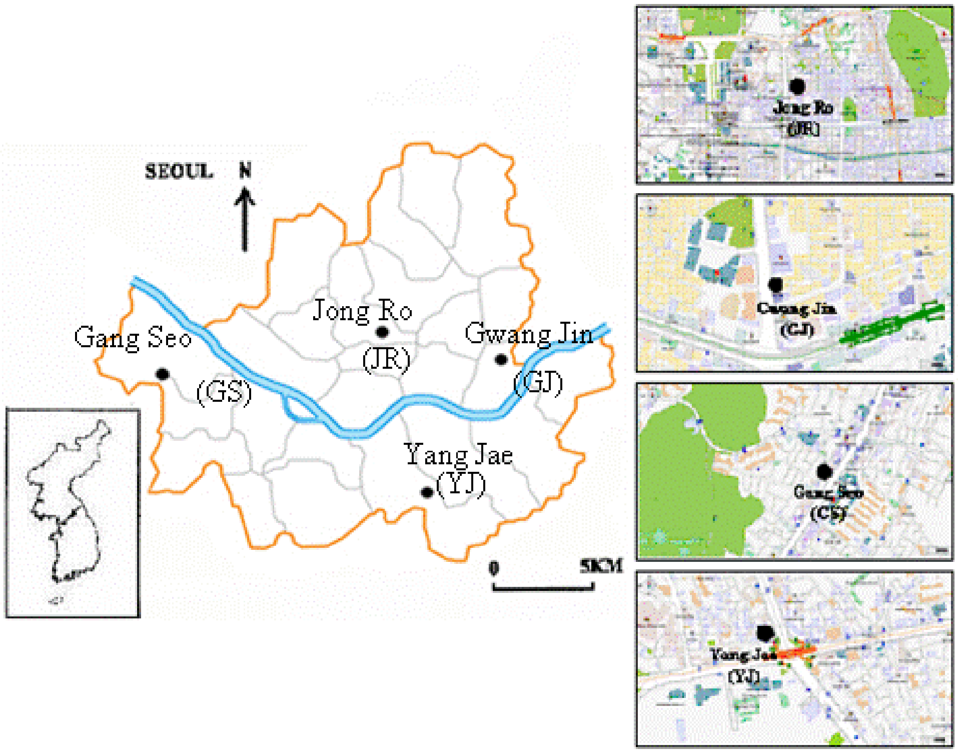

**FIGURE 1.** Geographical location of four study sites: Jong Ro (JR), Gwang Jin (GJ), Gang Seo (GS), and Yang Jae (YJ) in Seoul, Korea.

The metal elements in the TSP samples were analyzed with a pretreatment of 3.8-cm punching portions. The filter samples were pretreated with 5 mL of mixed acid (5.55% $HNO_3$/16.75% HCl), then 5 mL of 10 ppm $Y_2O_3$ was added as surrogate standards. Filter samples were placed in PFA vessels containing 10 mL of the above acid solution and then heated using a microwave digestion system (Tekton, Q45) to 180°C for 10 min. Extracted solutions were filtrated and washed by 5 mL of the above mixing acid to produce a final volume of 25 mL. Eventually, the metallic components were analyzed by the ICP system (Spectro DE/CIROS Vision, Germany). The instrumental settings used in the ICP analysis were as follows: plasma power = 1400 W; auxiliary flow = 1.0 L $min^{-1}$; nebulized flow = 1.0 L $ml^{-1}$; and coolant flow = 12.0 L $ml^{-1}$. The detection limits (DL) of all metals were typically found in the following ranges: (1) less than 1 µg (in absolute mass) or 0.1–0.5 ng $m^{-3}$ (Cd, V, Co, Ag, Sr, Mn, and Mo); (2) 1–5 µg or 1–3 ng $m^{-3}$ (Pb, Li, Ni, Cu, Ba, and As); (3) 5–7 µg or ~5 ng $m^{-3}$ (Cr and B); and (4) 16–24 µg or 14–21 ng $m^{-3}$ (Zn and Fe). Moreover, the accuracy of the metal analysis, if determined with reference to Accustandard (Cat. No: MES-06-1), was found in the following range: (1) less than 100% (Li, Ag, and Cu), (2) 100–110% (As, Sr, V, Zn, Mo, Ba, Co, Cd, Pb, Mn, Ni, Cr, and B), and (3) 112% (Fe). The precision of all metals was computed in the following range: (1) less than 1.0% (V, Mo, Co, Cd, Ag, As, and Mn), (2) 1–3% (Sr, Li, Zn, Ni, B, Ba, and Pb), and (3) 3–5% (Cu, Fe, and Cr).

## RESULTS AND DISCUSSION

### The Overall Picture of Metals at the Four Study Sites

As shown in Table 1, the concentrations of 17 metal species (Fe, Zn, Cu, Ba, Mn, Pb, Ag, As, B, Cd, Co, Cr, Li, Mo, Ni, Sr, and V) bound with the TSP samples have been monitored at four air quality monitoring

**TABLE 1**
**Concentration of Metal Species Measured at Four Sites in Seoul, Korea from February to July 2009[a]**

| | Jong Ro (JR) | Gwang Jin (GJ) | Gang Seo (GS) | Yang Jae (YJ) |
|---|---|---|---|---|
| TSP[b] | 125 ± 57.1 (113.9)[c] | 137.4 ± 80.9 (118.4) | 119.1 ± 67.1 (102.1) | 124.4 ± 57.9 (122.5) |
| | 48–237.7 (18)[d] | 45.5–325.7 (18) | 41.7–264.6 (18) | 37.4–247.4 (18) |
| Fe | 2599 ± 1513 (2427) | 2914 ± 2704 (2241) | 2685 ± 1863 (2358) | 2897 ± 1516 (2712) |
| | 462.9–5672 (18) | 259–12071 (18) | 444.1–8134 (18) | 625–6401 (18) |
| Zn | 210.8 ± 116.1 (171.6) | 436.5 ± 1047 (171.3) | 226 ± 177 (164) | 234.6 ± 167.2 (181.9) |
| | 61.7–455.9 (18) | 75.6–4614 (18) | 91.9–767.3 (18) | 65.4–791 (18) |
| Cu | 54.3 ± 33.0 (44.6) | 64.3 ± 56.5 (43.4) | 56.6 ± 41.8 (44.7) | 72.1 ± 49.2 (54.7) |
| | 21.8–126.1 (18) | 21.1–260.2 (18) | 20.9–175.3 (18) | 22.3–197.8 (18) |
| Ba | 80.7 ± 43.1 (76.6) | 68.8 ± 43.3 (52.5) | 74.2 ± 43.9 (67.2) | 90.9 ± 50.4 (76.8) |
| | 19.4–180.1 (18) | 16.6–170.6 (18) | 17.7–195.7 (18) | 28.6–221.4 (18) |
| Mn | 72.9 ± 41.0 (71.9) | 66.3 ± 38.5 (63.0) | 76.0 ± 53.8 (65.4) | 75.4 ± 37.7 (78) |
| | 12.9–161.9 (18) | 1.33–137.7 (18) | 17.5–238.5 (18) | 18.6–169.8 (18) |
| Pb | 64.4 ± 47.8 (50.2) | 59.2 ± 37.1 (44.3) | 69.3 ± 60.6 (44.5) | 77.2 ± 42.1 (66.0) |
| | 13.2–193.5 (18) | 7.5–132.5 (18) | 20.5–238.2 (18) | 18.3–173.0 (18) |
| Ag | 0.66 ± 0.24 (0.64) | — | — | — |
| | 0.43–0.91 (3) | 1.11–1.11 (1) | — | — |
| As | 5.73 ± 3.19 (4.47) | 6.00 ± 3.48 (5.33) | 6.26 ± 2.44 (5.74) | 7.61 ± 4.39 (6.52) |
| | 0.07–12.29 (15) | 2.73–15.11 (12) | 3.89–12.26 (12) | 3.08–17.6 (15) |
| B | 16.5 ± 11.7 (10.6) | 24.2 ± 14.5 (20.1) | 19.1 ± 9.94 (17.4) | 20.1 ± 8.33 (18.4) |
| | 6.82–44.4 (13) | 8.53–59.7 (12) | 5.58–44 (16) | 7.31–33.7 (17) |
| Cd | 2.33 ± 1.54 (1.96) | 2.54 ± 1.31 (2.3) | 2.62 ± 2.09 (1.9) | 2.42 ± 1.37 (1.96) |
| | 0.27–6.14 (18) | 0.74–5.24 (18) | 0.66–9.03 (18) | 0.83–6.31 (18) |
| Co | 1.66 ± 0.87 (1.44) | 1.36 ± 0.76 (1.35) | 1.57 ± 1.01 (1.38) | 1.59 ± 0.73 (1.53) |
| | 0.42–3.73 (18) | 0.25–2.98 (18) | 0.26–4.32 (18) | 0.49–3.42 (18) |
| Cr | 13.6 ± 5.47 (13.4) | 10.9 ± 5.05 (10.52) | 14.5 ± 12.7 (10.7) | 11.1 ± 4.10 (10.1) |
| | 7.29–24.8 (15) | 2.88–20.6 (16) | 5.79–56.6 (16) | 5.87–22.4 (17) |
| Li | 3.58 ± 1.63 (3.16) | 3.66 ± 1.7 (3.55) | 4.00 ± 2.53 (3.22) | 3.99 ± 2.01 (3.56) |
| | 1.52–6.93 (13) | 1.41–7.12 (13) | 1.47–10.1 (12) | 1.72–8.86 (15) |
| Mo | 2.54 ± 1.65 (2.29) | 2.45 ± 1.62 (2.00) | 2.25 ± 1.60 (2.00) | 2.53 ± 1.36 (2.15) |
| | 0.88–7.77 (18) | 0.52–6.13 (17) | 0.68–7.34 (18) | 0.78–6.26 (18) |
| Ni | 13.4 ± 18.2 (8.92) | 7.80 ± 3.96 (6.76) | 9.29 ± 6.65 (6.65) | 7.86 ± 3.81 (6.93) |
| | 4.17–83.2 (18) | 1.43–14.9 (18) | 3.49–27.2 (18) | 3.58–18.1 (18) |
| Sr | 12.3 ± 7.56 (12.1) | 11.7 ± 9.56 (9.86) | 12.2 ± 8.45 (11.4) | 12.5 ± 7.30 (13.2) |
| | 1.26–25.5 (18) | 1.82–35.1 (18) | 2.55–32.9 (18) | 1.92–28.1 (18) |
| V | 8.27 ± 5.3 (6.53) | 8.00 ± 5.13 (6.24) | 9.51 ± 7.3 (7.22) | 8.51 ± 5.29 (6.53) |
| | 1.32–20.1 (18) | 2.84–19.1 (18) | 2.30–29.2 (18) | 1.93–22.9 (18) |

[a]Metallic species (units in ng $m^{-3}$); [b]TSP in µg $m^{-3}$; [c]mean ± SD (median); [d]range (number of data).

sites representing residential areas in Seoul, Korea: Jong Ro (JR), Gwang Jin (GJ), Gang Seo (GS), and Yang Jae (YJ). To facilitate the interpretation of these metal data, the relevant meteorological parameters (e.g., TEMP, HUM, WS, WD, UV, and solar) and criteria pollutants (e.g., $SO_2$, NO, $NO_2$, $NO_x$, CO, $O_3$, $PM_{10}$, $PM_{2.5}$, $CH_4$, $NCH_4$, and THC) were also measured concurrently. A comparison of the mean wind speed data at the four sites indicated that the strongest wind occurred from the GJ site (2.04 ± 0.46 m $sec^{-1}$) and then decreased on the order of JR (1.35 ± 0.33), GS (1.07 ± 0.10), and YJ (0.90 ± 0.22 m $sec^{-1}$) (Table 2). The frequency distribution of wind rose data (Fig. 2) showed that winds at the JR and GJ sites occurred dominantly from the west with a relative frequency of around 22 and 59%, respectively. In contrast, the patterns of the GS and YJ sites changed considerably with the dominance of southerly (33%) and northwesterly winds (34%), respectively.

According to a statistical summary of the data, the concentration levels of different metals vary across several orders of magnitude (Table 1). If the sum values of all these metal components are computed in relation to the mass of TSP for each site, the relative fraction of these metal components are found to comprise 2–3% of the total particles. To make a comparison of all the metal concentration data on a comparable scale, the mean values for each metal are also plotted on a logarithmic scale (Fig. 3). The relative ordering of the metal concentration levels appears to be fairly consistent between different sites, as Fe consistently records the highest concentration of all four sites. The metal data at JR can be organized on the order of Fe > Zn > Ba > Mn > Pb > Cu > B > Cr > Ni > Sr >V > As > Li > Cd > Mo > Co > Ag. Likewise, the patterns for the other sites are highly comparable with that of JR, while there were slight alterations in their order at other sites, such as between Mn and Ba or between Sr and N.

## Comparison of Observed Metal Levels Across the Four Sites

In light of the fact that our measurements were made from several locations within the city boundaries of Seoul, our metal data can be used to evaluate the role of spatial factors on metal distribution characteristics. In order to estimate the pollution status of metals across all four study sites, the mean values from different sites were compared in terms of the absolute magnitude. As seen in Table 1, the mean TSP concentrations fell in a relatively narrow range of 119.1 ± 67.1 (GS) to 137.4 ± 80.9 μg $m^{-3}$ (GJ site).

According to a comparison of the metal concentration levels between the four sites, the highest values were typically seen from Fe (JR: 2599 ± 1513; GJ: 2914 ± 2704; GS: 2685 ± 1863; and YJ: 2897 ± 1516 ng $m^{-3}$). In contrast, the lowest values were dominated by either Ag (JR: 0.66 ± 0.24 and GJ: 1.11 ng $m^{-3}$) or Co (GS: 1.57 ± 1.01 and 1.59 ± 0.73 ng $m^{-3}$). The predominance of Fe in urban areas is in fact not uncommon, as frequently seen previously in numerous studies (e.g., [11,17,20,21,22]). This can be ascribed to the contribution of resuspended soil/road dust[23], in association with traffic activities in high-traffic urban environments[20,24]. The mean concentration levels of Zn, seen in the range of 210.8 (JR) to 436.5 (GJ) ng $m^{-3}$, were approximately one order of magnitude lower than Fe. The concentrations of Cu, Ba, Mn, and Pb, arising from the combustion of fossil fuels[6], fell in the range between 50 and 90 ng $m^{-3}$. Pacyna and Pacyna[6] reported that the largest emissions of Pb occurred due to the combustion of leaded gasoline. In Mumbai, India, about 60% of Pb was due to vehicular emissions at a traffic junction site, while 20% of that was accounted for by road dust[12]. Other metals (e.g., As, B, Cd, Co, Cr, Li, Mo, Ni, Sr, and V) showed much reduced levels (e.g., mean values of less than 25 ng $m^{-3}$). In fact, previous studies in Seoul suggested that the high concentrations of Zn, Cu, and Pb were affected most strongly by such anthropogenic inputs as fossil fuel, steel tool houses, and machine shops[19].

The normalization of these metal concentration data also indicates that the highest concentrations of metal data were seen most commonly from the GJ and YJ sites, as both sites recorded the max concentrations of four out of 17 metals. In contrast, the lowest values are seen most frequently at the JR site (eight out of 17 metals). This relative dominance at the YJ site has also been recognized in the analysis of the PAH levels that were simultaneously measured during our study[25]. The findings of relative enhancement at the YJ site are suspected to originate from the outdoor incineration activities of small flower-planting facilities dispersed throughout the YJ area.

**TABLE 2**
**Summary of Relevant Parameters Measured at Four Study Sites in Seoul, Korea from February to July 2009**

| | | Jong Ro (JR) | Gwang Jin (GJ) | Gang Seo (GS) | Yang Jae (YJ) |
|---|---|---|---|---|---|
| *A. Criteria pollutants* | | | | | |
| $SO_2$ | (ppb) | 7.83 ± 3.167 (7)[a] | 4.28 ± 2.54 (4) | 7.72 ± 4.31 (6) | 5.06 ± 3.44 (4) |
| | | 5–16 (18)[b] | 2–10 (18) | 5–21 (18) | 2–14 (18) |
| NO | (ppb) | 15.61 ± 11.64 (11) | 17.1 ± 13.8 (13.5) | 15.9 ± 16 (9.5) | 21.7 ± 17.2 (16) |
| | | 3–41 (18) | 4–50 (18) | 3–56 (18) | 3–55 (18) |
| $NO_2$ | (ppb) | 41.3 ± 17.4 (35.5) | 40.8 ± 15.5 (37.5) | 39.7 ± 18 (32.5) | 41.8 ± 16.6 (38.5) |
| | | 24–79 (18) | 22–75 (18) | 18–80 (18) | 17–84 (18) |
| NOx | (ppb) | 57.2 ± 27 (46) | 57.6 ± 27.4 (47) | 55.4 ± 31.9 (41) | 63.2 ± 31.2 (54.5) |
| | | 27–113 (18) | 25–113 (18) | 21–123 (18) | 21–127 (18) |
| CO | (ppb) | 772 ± 267 (700) | 750 ± 182.3 (700) | 516.7 ± 206.5 (450) | 600 ± 208.6 (550) |
| | | 500–1300 (18) | 500–1100 (18) | 200–900 (18) | 300–1100 (18) |
| $O_3$ | (ppb) | 23.5 ± 9.71 (24) | 27.7 ± 12.4 (26.5) | 26.3 ± 11.9 (27) | 22.6 ± 11.9 (20) |
| | | 9–47 (18) | 10–55 (18) | 5–50 (18) | 9–48 (18) |
| $PM_{10}$ | (μg $m^{-3}$) | 61.9 ± 29.1 (51.5) | 58.8 ± 29.5 (50) | 65.2 ± 38.5 (54.5) | 62.4 ± 33.7 (54) |
| | | 32–126 (18) | 26–127 (18) | 24–151 (18) | 27–145 (18) |
| $PM_{2.5}$ | (μg $m^{-3}$) | 27.9 ± 13.4 (24.5) | 29.7 ± 14.5 (24) | 32.2 ± 18.9 (25.5) | 30.1 ± 18.6 (22.5) |
| | | 13–59 (18) | 16–62 (18) | 16–72 (18) | 15–74 (18) |
| $CH_4$ | (ppmC) | 2.23 ± 0.35 (2.26) | 1.99 ± 0.14 (2.01) | 2.13 ± 0.13 (2.15) | 2.09 ± 0.15 (2.05) |
| | | 1.79–2.83 (18) | 1.74–2.26 (18) | 1.93–2.43 (18) | 1.93–2.43 (18) |
| $NCH_4$ | (ppmC) | 0.32 ± 0.07 (0.3) | 0.25 ± 0.11 (0.25) | 0.34 ± 0.05 (0.35) | 0.28 ± 0.09 (0.28) |
| | | 0.26–0.51 (18) | 0.09–0.49 (18) | 0.25–0.42 (18) | 0.16–0.48 (18) |
| THC | (ppmC) | 2.55 ± 0.37 (2.56) | 2.24 ± 0.22 (2.22) | 2.47 ± 0.15 (2.5) | 2.38 ± 0.23 (2.36) |
| | | 2.06–3.18 (18) | 1.89–2.6 (18) | 2.25–2.78 (18) | 2.09–2.91 (18) |
| *B. Meteorological parameters* | | | | | |
| TEMP | ($^{o}$C) | 22.8 ± 8.09 (22.4) | 19.0 ± 7.75 (21.25) | 18.9 ± 7.33 (20.5) | 22.7 ± 6.77 (24.8) |
| | | 7.8–36.4 (18/18) | 5.1–31.6 (18/18) | 7.4–30.5 (18/18) | 9.5–35 (18/18) |
| HUM | (%) | 53.2 ± 19.4 (47.5) | 52.8 ± 19.2 (45.5) | 52.1 ± 16.2 (47) | 50.8 ± 16.6 (45) |
| | | 28–89 (18) | 28–84 (18) | 32–85 (18) | 29–82 (18) |
| WS | (m $sec^{-1}$) | 1.35 ± 0.33 (1.3) | 2.04 ± 0.46 (1.95) | 1.07 ± 0.1 (1.1) | 0.9 ± 0.22 (0.85) |
| | | 0.8–2.1 (18) | 1.1–3.1 (18) | 0.9–1.3 (18) | 0.6–1.3 (18) |
| UV | (mW $cm^{-2}$) | 0.49 ± 0.27 (0.42) | 0.43 ± 0.19 (0.43) | 0.36 ± 0.18 (0.32) | 0.4 ± 0.22 (0.35) |
| | | 0.12–1.07 (18) | 0.09–0.76 (18) | 0.08–0.72 (18) | 0.1–0.85 (18) |
| Solar | (W $m^{-3}$) | 180.7 ± 78.6 (192) | 158.1 ± 75.2 (168) | 172.3 ± 75.7 (185.5) | 163.5 ± 72.3 (172.5) |
| | | 37–300 (18) | 22–280 (18) | 43–292 (18) | 47–270 (18) |

[a]Mean ± SD (median); [b]range (number of data).

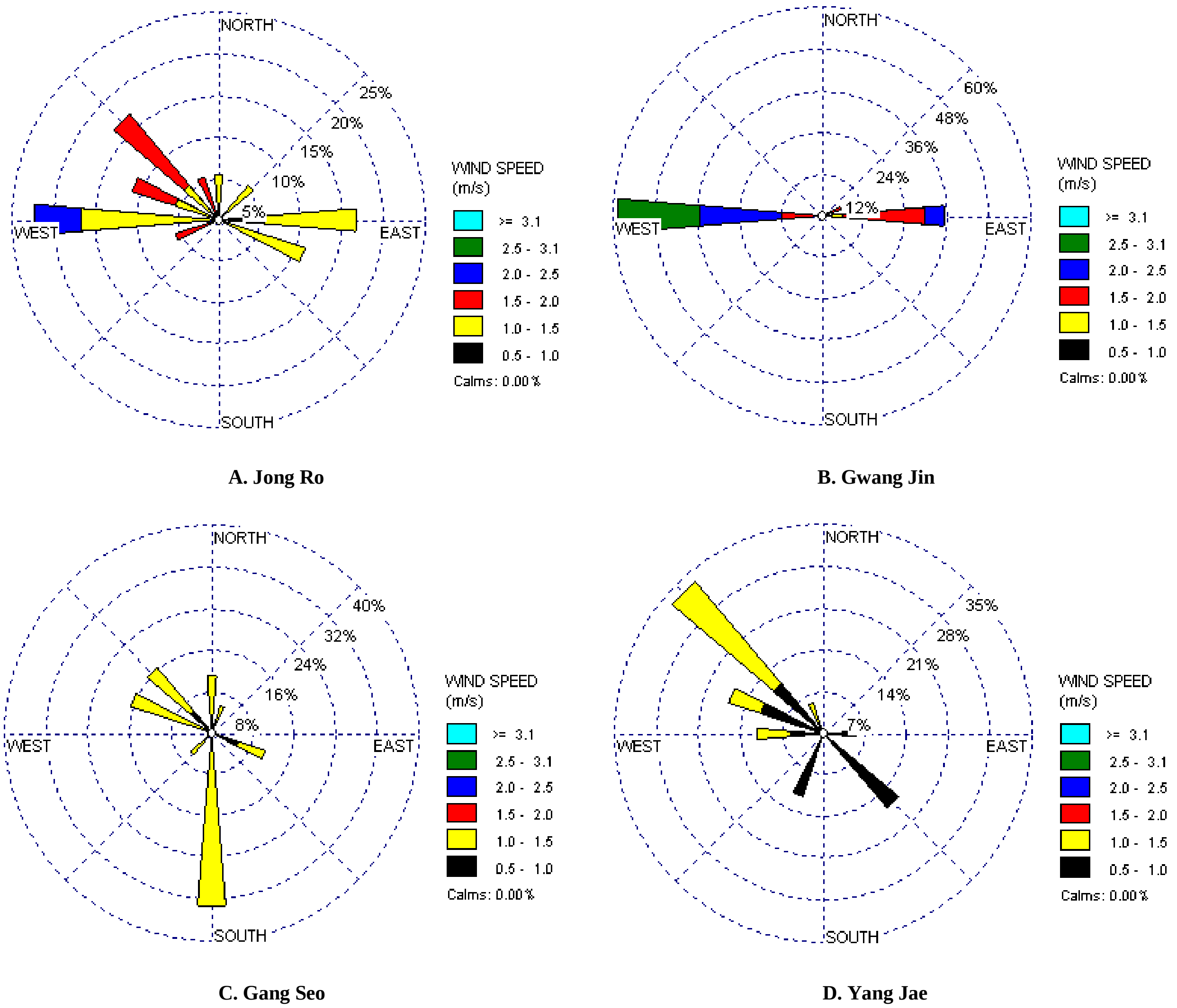

**FIGURE 2.** Frequency distribution of wind rose patterns at the four study sites during the study period (February to July 2009).

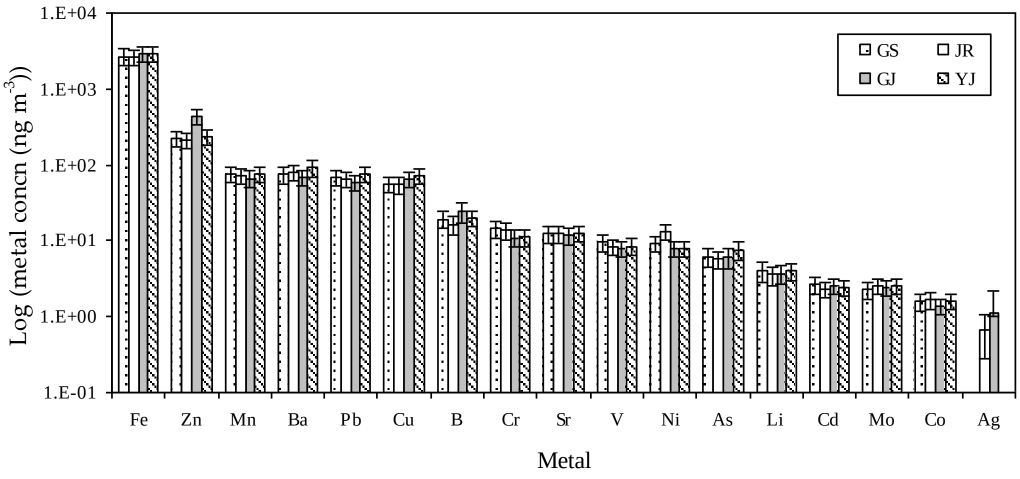

**FIGURE 3.** Comparison of the mean metal concentration in TSP across the four study sites.

To examine the possible effect of local sources on the observed metal levels, the distribution of TSP data was plotted in relation to the concurrently measured wind rose pattern. As shown in Fig 4, the enhanced TSP value of 164.7 μg $m^{-3}$ was seen in the western sector at GJ (59% of wind rose), followed by the eastern sector (35% of wind rose) of 98.0 μg $m^{-3}$. As the GJ monitoring site is located in the east side of the city at a down-wind position from the busy trafficking highway, it is apt to be affected by local sources. In contrast, the highest concentration of TSP (237.7 μg $m^{-3}$) at the JR site occurred from the north, although wind occurrence at the site was dominated from the west.

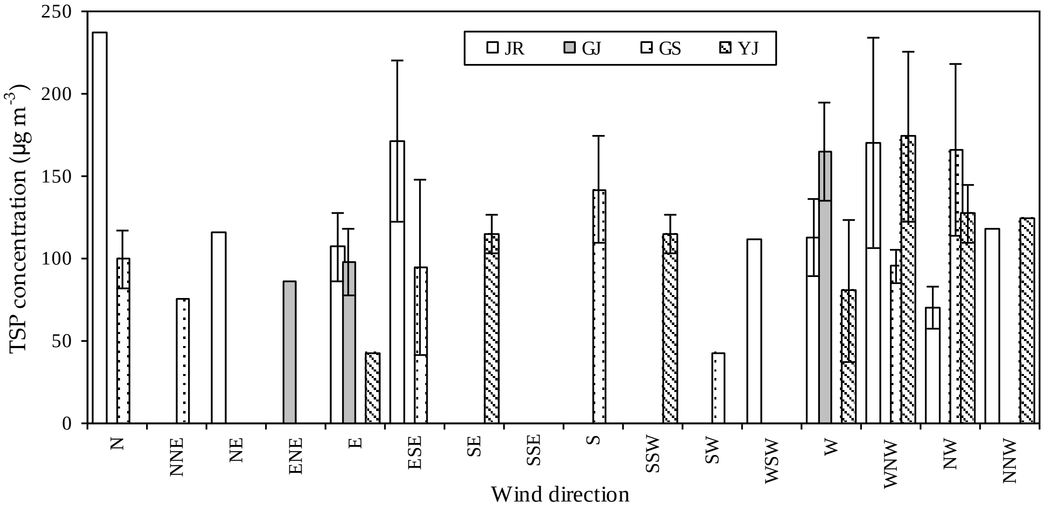

**FIGURE 4.** Comparison of the TSP concentration levels in relation to wind sectors (1 SD value used for error bar).

As a simple means to assess the relative contribution of anthropogenic vs. crustal sources, many previous studies have applied the calculation of enrichment factor (EF) for the particle-bound metals (e.g., [22,26,27]). As the concept of EF is derived relative to well-defined crustal sources (such as Fe, Al, and Mn), such values can be differentiated between different metals of diverse source processes[28]. As Fe is one of the most commonly used reference elements, EF based on this reference has commonly been used for a variety of compartments (e.g., [29]). If such a principle is applied to our data, the extent of enrichment (or depletion) for a given metal can be computed by the following equation:

$$EF = \frac{\left\{\frac{X}{Fe}\right\}_{sample}}{\left\{\frac{X}{Fe}\right\}_{crust}}$$, where X denotes a metal of interest.

As seen in Table 3, the results of EF derived at the four sites are compared by assuming the EF value of 10 as the cut-off criterion for excess enrichment. Hence, excessive enrichment was seen most dominantly from such metals as: (1) Ag, Ac, and Pb (JR); (2) Ag, As, Cd, Pb, and Zn (GJ); (3) Cd and Pb (GS); and (4) As, Cd, and Pb (YJ). In contrast, the lowest EF values ( <10) at all four sites were generally recorded by such metals as Ba, Co, Cr, Li, Mn, Ni, Sr, and V. The large difference between EF values is consistently observed in different metals with high (Pb, Cd, and Zn) and low EF values (Mn, Cr, and Ni) at all four study sites. This pattern in urban areas has been commonly observed in previous studies[21]. The results of the EF computation thus indicate that the metal pollution in these study areas should effectively reflect man-made activities including traffic sources.

**TABLE 3**
**Statistical Summary of EF Values Derived using Metal Concentration of Four Sites in Seoul[a]**

| | EF[b] (JR) | | | EF (GJ) | | | EF (GS) | | | EF (YJ) | | |
|---|---|---|---|---|---|---|---|---|---|---|---|---|
| | Mean | SD | n | Mean | SD | n | Mean | SD | n | Mean | SD | n |
| Ag | 412 | 64.1 | 3 | 466 | | 1 | | | | | | |
| As | 97.1 | 68.5 | 15 | 143 | 169 | 12 | 88.9 | 58.3 | 12 | 102 | 64.0 | 15 |
| B | 27.8 | 12.0 | 13 | 59.2 | 68.9 | 12 | 43.3 | 27.7 | 16 | 47.8 | 28.1 | 17 |
| Ba | 4.57 | 1.40 | 18 | 4.28 | 1.89 | 18 | 4.09 | 1.22 | 18 | 4.40 | 1.37 | 18 |
| Cd | 256 | 73.8 | 18 | 459 | 721 | 18 | 301 | 143 | 18 | 251 | 77.9 | 18 |
| Co | 1.62 | 0.67 | 18 | 1.46 | 0.99 | 18 | 1.35 | 0.15 | 18 | 1.29 | 0.17 | 18 |
| Cr | 2.75 | 1.08 | 15 | 2.62 | 1.36 | 16 | 3.04 | 1.55 | 16 | 2.22 | 0.57 | 17 |
| Cu | 24.9 | 11.4 | 18 | 40.7 | 49.6 | 18 | 25.4 | 13.0 | 18 | 28.0 | 15.9 | 18 |
| Li | 3.04 | 0.54 | 13 | 6.94 | 11.7 | 13 | 3.09 | 0.55 | 12 | 3.41 | 0.95 | 15 |
| Mn | 1.68 | 0.17 | 18 | 1.56 | 0.51 | 18 | 1.70 | 0.22 | 18 | 1.57 | 0.17 | 18 |
| Mo | 43.2 | 19.9 | 18 | 86.2 | 207 | 17 | 36.9 | 19.5 | 18 | 37.5 | 18.8 | 18 |
| Ni | 4.71 | 5.87 | 18 | 2.97 | 1.89 | 18 | 3.06 | 1.74 | 18 | 2.38 | 1.21 | 18 |
| Pb | 109 | 39.8 | 18 | 119 | 61.8 | 18 | 120 | 58.1 | 18 | 121 | 37.6 | 18 |
| Sr | 0.68 | 0.16 | 18 | 0.74 | 0.35 | 17 | 0.67 | 0.1 | 18 | 0.62 | 0.13 | 18 |
| V | 1.47 | 0.92 | 18 | 1.83 | 1.48 | 18 | 1.80 | 1.6 | 18 | 1.25 | 0.35 | 18 |
| Zn | 80.4 | 60.5 | 18 | 155 | 305 | 18 | 75.0 | 32.2 | 18 | 70.0 | 39.3 | 18 |

[a]Fe is used as a reference metal for EF calculation; [b]EF is computed by the formula noted in text[22].

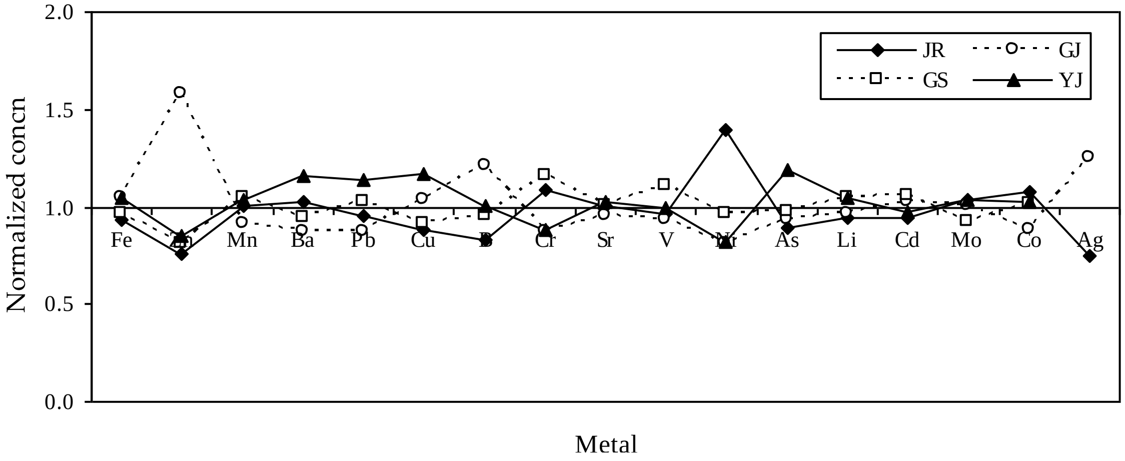

**FIGURE 5.** Normalization of the mean metal concentration in TSP among the four sites in this study.

As seen in Fig. 5, the metal concentration data for each site are also compared after normalization by their respective mean values at each site. Accordingly, the largest variation is seen in the case of Zn, which is discernible from others like Fe, Sr, Li, Cd, and Mo. Moreover, if the coefficient of variation (CV: the ratio between the standard deviation and mean) for each metal (except Ag with limited data) is compared across the four study sites, its magnitude is found in the ascending order of Fe < Sr < Cd < Mo < Li < Mn < V < Co < Pb < Ba <Cu < As < Cr < B < Ni < Zn. The largest CV value at the four sites was seen on the order of Zn (with 38.6%) and Ni (with 27.6%), while the next six elements (from B to Pb) had

CV values in the 10–20% range. In contrast, the CV values for the lowest eight metals were below 10%. The large spatial variability of Zn, as shown in terms of CV, may be accounted for by the complicated interaction between its chemical properties and the emission sources. Although it is difficult to account for the variability, its effect is also likely reflected in the unusually large EF values. Bilos et al.[21] demonstrated that a high EF of Zn (~366) at a residential site in La Plata city, Argentina was associated with the activities of high-pollutant–emitting vehicles. In an urban area of Stockholm, Sweden, major Zn sources were also estimated to be the resuspension of road dust, while around 40% of road traffic emissions of Zn are allocated to exhaust emissions[30].

## The Temporal Variability of Metal Distribution

To analyze the temporal variability of metal species measured from all study sites, their seasonal distribution is compared through normalization of seasonal mean values. If the 6-month period (February to July, 2009) is divided into winter, spring, and summer, the relative dominance of individual metal data from each site was recognized most frequently in spring (except for As). As shown in Fig. 6, a comparison of seasonal patterns of each metal is facilitated through the normalization of their seasonal mean values. If the results are compared by daily measurement values, such patterns are clearly distinct. At the GS site, the highest daily values of Mn and Pb were seen on April 27 as 239 and 238 ng $m^{-3}$, respectively. Likewise, their peak values (April 28) at YJ were observed at 137.7 and 132.5 ng $m^{-3}$, respectively. However, the As data showed the maximum daily value in February such as 12.3 (GS), 15.1 (GJ), and 17.6 (YJ) on February 24. In the case of Ni, its maximum daily value was also observed on spring days at or below 27.2 ng $m^{-3}$ (except the JR site). Tsai et al.[31] suggested that the wintertime peak of As, observed from Tainan, Taiwan, may be explained by its stronger source activities under cold-weather conditions with low mixing depth.

The occurrences of high metal concentration levels in springtime can be explained by the effects of Asian Dust (AD) events. It was demonstrated that the enrichment of heavy metals in urban locations was affected by the long-range transport of airborne pollutants (e.g., northern China during springtime)[32]. Based on comparisons of particle-bound metal levels between AD and non-AD periods, Kim and Kim[33] also showed significantly enhanced TSP concentrations during AD (410 µg $m^{-3}$) relative to non-AD (169 µg $m^{-3}$) in a residential area of Seoul. Moreover, Han et al.[34] reported that the average Pb flux during AD (199 µg $m^{-2}$ $day^{-1}$) was about 4.7 times higher than during non-AD. As such, the occurrences of the AD event can exert direct influences on the seasonal distribution of TSP and metals in urban areas (e.g., [35]).

Note that the lowest concentration of TSP and metals in the summer months may be ascribable to the effect of the summertime monsoon and the development of deep mixing layers[36,37,38]. The mean precipitation amounts in May and June ranged from 13.2 (YJ) to 21.7 mm (GS), although there was no precipitation from February to April during our measurements.

## Factors Affecting the Environmental Behavior of Metals

To estimate the basic factors and processes affecting the metal distribution at all four sites, the concentration data of different metals and the relevant parameters were examined with the aid of a factor analysis (FA). As part of principal component analysis (PCA), FA is based on a numerical approach to account for the statistical variance of the data by producing a small number of factors from a large number of variables[39]. In this study, FA was preformed by the principle component method with eigenvalue criterion (>1.0) and a factor loading of 0.3 or higher to retain the factors for further evaluation (Table 4). Hence, the varimax rotated factor loading matrix was examined with respect to a small group of metals and the relevant parameters to identify similar source properties. Based on our FA analysis, up to three major components were identified for each site, which accounted for the total variance of 77.5% (YJ) to 88.3% (GJ) (Table 4). The first factor for the three sites was strongly associated with those metals

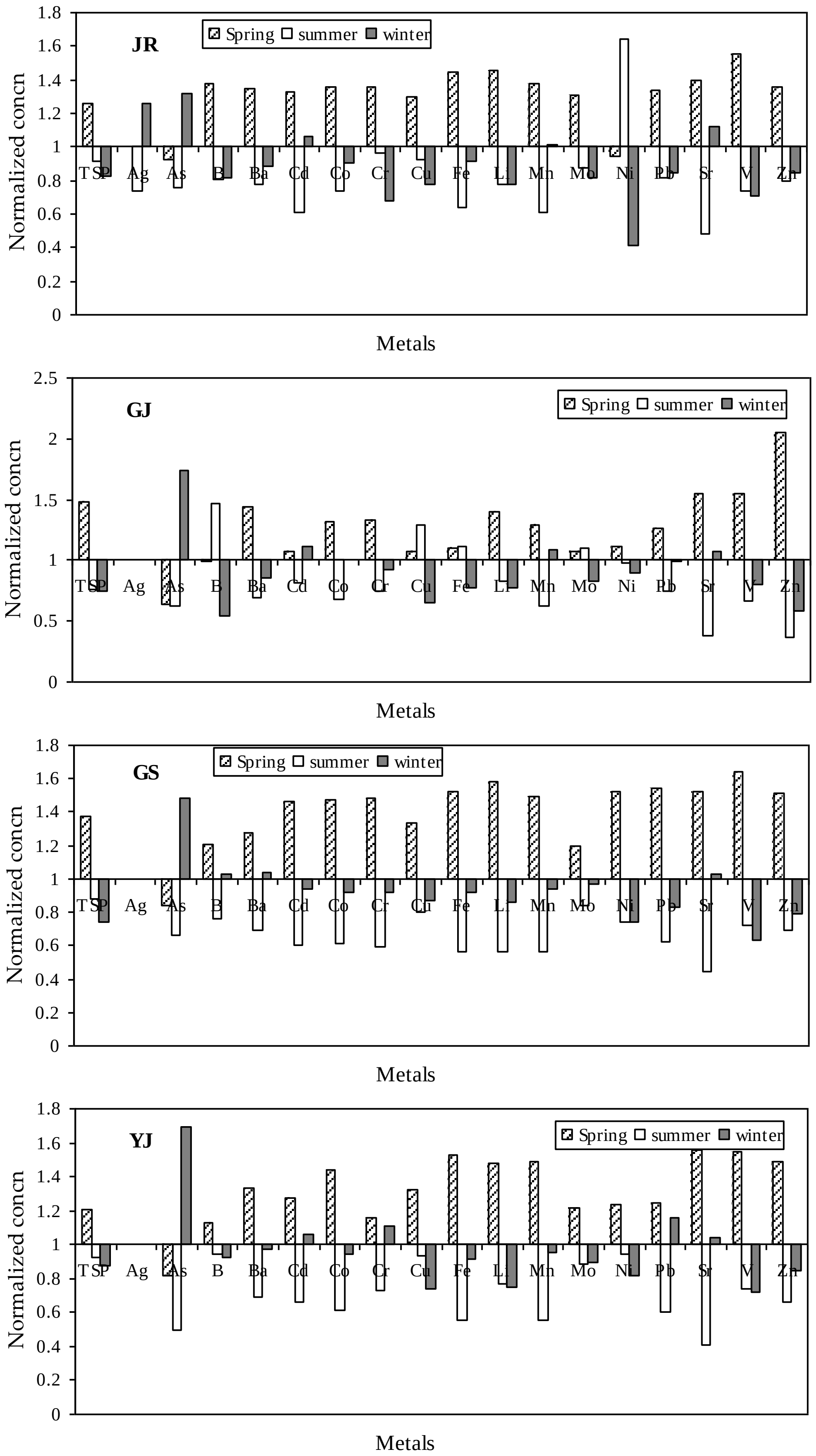

**FIGURE 6.** Normalization of metal species in TSP at each study site across seasons.

**TABLE 4**
**Results of Factor Analysis for Metals and Relevant Environmental Parameters at Each Station[a]**

| | JR | | | | GJ | | | | GS | | | | YJ | | | |
|---|---|---|---|---|---|---|---|---|---|---|---|---|---|---|---|---|
| | PC1[b] | PC2 | PC3 | Communalities | PC1 | PC2 | PC3 | Communalities | PC1 | PC2 | PC3 | Communalities | PC1 | PC2 | PC3 | Communalities |
| As | | | | 0.06 | | | –0.63 | 0.45 | | **0.88** | | 0.815 | | –0.52 | | 0.29 |
| B | **0.90** | | | 0.82 | | 0.61 | **0.72** | 0.94 | **0.75** | 0.39 | | 0.765 | 0.54 | 0.31 | | 0.42 |
| Ba | **0.92** | | | 0.87 | **0.78** | 0.44 | 0.34 | 0.91 | **0.93** | | 0.30 | 0.971 | **0.87** | | 0.38 | 0.90 |
| Cd | **0.88** | | | 0.83 | **0.78** | 0.48 | | 0.84 | **0.97** | | | 0.952 | **0.92** | | | 0.91 |
| Co | **0.75** | –0.44 | 0.34 | 0.87 | **0.73** | 0.66 | | 0.96 | **0.97** | | | 0.961 | **0.98** | | | 0.98 |
| Cr | 0.41 | | | 0.24 | 0.66 | **0.71** | | 0.95 | **0.95** | | | 0.973 | **0.95** | | | 0.93 |
| Cu | **0.93** | | | 0.89 | **0.83** | 0.34 | 0.45 | 1.00 | **0.93** | | | 0.895 | **0.81** | | 0.33 | 0.81 |
| Fe | **0.90** | | 0.34 | 0.94 | **0.71** | 0.67 | | 0.95 | **0.94** | | | 0.899 | **0.95** | | | 0.96 |
| Li | **0.86** | | 0.39 | 0.90 | 0.54 | 0.57 | 0.56 | 0.93 | **0.96** | | | 0.922 | **0.91** | 0.32 | | 0.94 |
| Mn | **0.87** | | 0.32 | 0.89 | **0.74** | 0.64 | | 0.99 | **0.93** | | | 0.899 | **0.97** | | | 0.96 |
| Mo | **0.77** | | | 0.68 | **0.81** | 0.35 | | 0.81 | **0.90** | | | 0.94 | **0.82** | | | 0.74 |
| Ni | | | –0.91 | 0.86 | 0.68 | 0.69 | | 0.94 | **0.99** | | | 0.98 | **0.83** | | | 0.77 |
| Pb | **0.87** | | –0.44 | 0.95 | 0.62 | **0.75** | | 0.95 | **0.98** | | | 0.979 | **0.85** | | 0.35 | 0.87 |
| Sr | **0.86** | | 0.44 | 0.93 | **0.80** | | | 0.66 | **0.96** | | | 0.937 | **0.89** | | | 0.87 |
| V | **0.95** | | | 0.98 | 0.53 | **0.80** | | 0.96 | **0.99** | | | 0.989 | **0.93** | 0.35 | | 0.99 |
| Zn | **0.92** | | | 0.85 | 0.44 | –0.66 | | 0.65 | **0.97** | | | 0.96 | 0.43 | | –0.42 | 0.42 |
| $SO_2$ | **0.84** | | | 0.84 | 0.49 | **0.84** | | 0.95 | **0.82** | | –0.37 | 0.811 | | **0.76** | | 0.67 |
| NO | | **0.74** | | 0.69 | **0.97** | | | 0.94 | **0.86** | | | 0.823 | | –0.34 | **0.90** | 0.95 |
| $NO_2$ | **0.83** | 0.33 | | 0.85 | **0.73** | 0.47 | 0.49 | 0.99 | **0.97** | | | 0.969 | 0.44 | | **0.83** | 0.92 |
| NOx | 0.65 | 0.54 | | 0.78 | **0.89** | | 0.30 | 0.97 | **0.95** | | | 0.951 | | | **0.94** | 0.98 |
| CO | **0.81** | 0.49 | | 0.91 | 0.38 | | **0.77** | 0.82 | 0.51 | 0.62 | –0.35 | 0.756 | 0.32 | 0.35 | **0.79** | 0.86 |
| $O_3$ | | –0.81 | | 0.78 | | 0.58 | **0.75** | 0.98 | | –0.83 | –0.48 | 0.947 | | **0.83** | –0.37 | 0.89 |
| TSP | **0.94** | | | 0.92 | 0.54 | **0.83** | | 1.00 | **0.96** | | | 0.93 | 0.33 | **0.86** | 0.34 | 0.96 |
| $PM_{10}$ | **0.89** | | 0.33 | 0.92 | 0.57 | **0.79** | | 0.98 | **0.95** | | | 0.98 | 0.52 | 0.66 | 0.36 | 0.83 |
| $PM_{2.5}$ | **0.95** | | | 0.91 | 0.41 | **0.85** | 0.34 | 1.00 | **0.92** | | | 0.931 | | **0.85** | 0.36 | 0.89 |
| $CH_4$ | | **0.74** | 0.45 | 0.75 | | | **0.96** | 0.98 | 0.43 | | 0.49 | 0.428 | | 0.36 | **0.74** | 0.68 |
| $NCH_4$ | 0.49 | **0.73** | | 0.82 | 0.59 | 0.30 | **0.74** | 0.97 | **0.73** | 0.30 | | 0.627 | | | **0.89** | 0.89 |
| THC | | **0.78** | 0.44 | 0.81 | 0.32 | | **0.95** | 1.00 | 0.61 | | 0.38 | 0.53 | | 0.31 | **0.81** | 0.77 |
| TEMP | 0.57 | –0.44 | –0.37 | 0.65 | | | 0.94 | 0.94 | | –0.57 | **0.73** | 0.934 | 0.32 | 0.45 | | 0.31 |
| HUM | | | –0.89 | 0.80 | –0.74 | | 0.35 | 0.73 | –0.32 | | 0.67 | 0.593 | –0.48 | | | 0.28 |
| WS | | –0.79 | 0.35 | 0.82 | | 0.58 | –0.72 | 0.91 | –0.64 | –0.54 | | 0.76 | | | –0.65 | 0.46 |
| UV | | –0.88 | | 0.82 | 0.41 | 0.74 | | 0.75 | –0.35 | –0.86 | | 0.897 | 0.79 | 0.37 | | 0.84 |
| Solar | | –0.73 | 0.59 | 0.97 | 0.44 | 0.75 | | 0.80 | | –0.84 | | 0.78 | **0.84** | 0.35 | | 0.87 |
| Eigenvalues | 24.7 | 7.70 | 4.38 | | 27.3 | 7.23 | 5.23 | | 29.9 | 5.09 | 3.67 | | 21.1 | 6.98 | 6.74 | |
| % of variance | 54.9 | 17.1 | 9.73 | 81.7 | 60.6 | 16.1 | 11.6 | 88.3 | 66.3 | 11.3 | 8.16 | 85.8 | 47.0 | 15.5 | 15.0 | 77.5 |

[a]Extraction method: PCA; rotation method: varimax with Kaiser normalization; [b]PC: Principle component loading.

originating from both resuspended soil/road dust (e.g., Fe and Mn) and the combustion of fossil fuel (e.g., Cu, Mn, Cd, and Zn) with a variance of 47.0 (YJ) to 66.3% (GS). The PM data with different fractions (such as $PM_{2.5}$ and $PM_{10}$) also belonged to the first factor at the JR and GS sites. On the other hand, the first factor (60%) at the GJ site suggested the dominant contribution of resuspended oil/road dust (e.g., Cu, Mo, and Sr), while burning coal and fuel oils (e.g., V and Pb) were the next important components with 16.1% of variance.

The dominant role of both resuspended soil/road dust and the combustion of fossil fuel as the source of metals has been postulated from numerous prior studies. For instance, Kumar et al.[12] reported that road dust and vehicular emissions contributed to more than 50% of the suspended PM at the Sakinaka traffic junction in Mumbai, India. Such processes are also known as the essential sources of Fe and Mn[23]. Moreover, combustion of oil is often referred to as the most effective source of Ni and V, while the iron and steel industry can also contribute to the build up of Cr and Mn[40].

In order to describe the relationship between metals and the relevant parameters, a correlation analysis was conducted further using the data sets collected at each station. To allow for a meaningful comparison of such analyses, the strengths of the correlation were compared after arbitrarily dividing the results into five classes: (1) no class (the weakest correlation strength range): $r \geq 10^{-1}$; (2) class I: $10^{-3} \leq r < 10^{-1}$; (3) class II: $10^{-5} \leq r < 10^{-3}$; (4) class III: $10^{-10} \leq r < 10^{-5}$; and (5) class IV (the

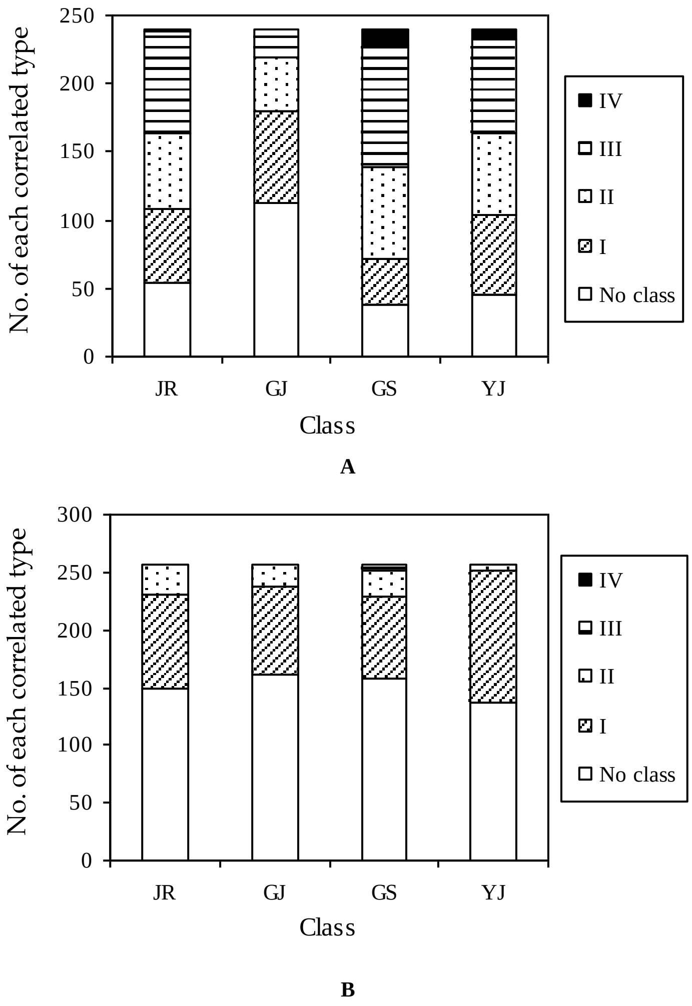

**FIGURE 7.** Relative abundance of the matching data pairs in terms of correlation strengths: (A) between different metals and (B) between metals and relevant parameters (criteria pollutants and meteorological parameters)

strongest correlation range): $r < 10^{-10}$. The results of this analysis, as summarized in Fig. 7, indicated that the strongest correlations tended to occur most abundantly at GS (class IV [n = 12] and III [n = 89]), which was then followed by YJ (class IV [n = 8] and class III [n = 68]), JR (class IV [n = 2] and class III [n = 74]), and GJ site (class III [n = 20]). According to this analysis, the strongest correlation pairs (class IV) tended to occur more frequently from metals existing at high concentration levels such as Fe (1 [JR]; 2 [GS]; and 3 [YJ]) and Mn (1 [JR]; 2 [GS]; and 3 [YJ]). The existence of strong correlations between metals of high concentrations was reported previously in many urban areas (e.g., [17]). In contrast, no such pairs were observed from metals with low concentration levels (e.g., As, Li, B, Ni, and Mo) at any of the four sites. These results further confirm that the intermetal relationships are highly pronounced relative to their pairs with relevant parameters. Moreover, although strongly correlated pairs were seen abundantly at the GS site, this was not the case for the GJ site. In addition, the strongly correlated cases between metals and relevant parameters (e.g., class II, III) are seen with the least abundance at the YJ site.

## Comparison with Previous Studies

To estimate the status of metal pollution at all study sites, the metal concentration data of this study were compared with those reported previously from various locations around the globe (Table 5). For this purpose, the data measured from urban areas were compiled to derive common patterns especially between commercial and residential areas. Considering the large differences in the spatial distribution of airborne metals, the metal concentration data were grouped on a geographical basis between Asian, European, and American countries. A comparison of these data indicated that the mean metal levels in Asian countries are approximately several-fold higher than those of the European and American countries with a few exceptions (e.g., [36,41]). The generally enhanced pollution levels in the Asian countries have been attributed to the consequences of poor control efforts on anthropogenic source processes, which is often accompanied by the massive consumption of anthracite coal (e.g., a heating fuel for residential facilities)[6].

As seen from numerous urban background areas in Asian countries, the highest values were recorded from Hok Tsui, Hong Kong, China[42] with the concentrations (ng $m^{-3}$) of 20,620 (Mn), 4970 (Cr), 8270 (Ni), and 3620 (As). Chan et al.[43] explained that the notably enhanced metal concentrations in Hong Kong may be affected by its topography, with very complex land use (a mixture of residential, commercial, and industrial areas) within the narrow boundary of the city. As high-rise buildings are close to each other, they may interfere with the dispersion of pollutant species[43]. A commercial and residential area in Kanpur, India is well known to have been affected by the major particulate sources, including heavy oil, coal, diesel in vehicles, soft coal for domestic cooking, and refuse burning[44]. The metal concentrations (ng $m^{-3}$) in Kanpur were also found in the considerably high range of 300–6170 (Fe), 200–1630 (Zn), 70–1030 (Pb), 2–43 (Cd), 32–400 (Cr), and 40–270 (Ni) (during 2002–2003)[44]. Except for Fe, Cu, Ba, and Mn, the metal concentrations in this study were about two to six times lower than those measured in Seoul during winter 2002[45]. As seen in Table 5, the mean Pb and Ni concentration levels at all four sites in the present study of 67.6 and 9.59 ng $m^{-3}$, respectively, are around three and five times less than 200 (Pb) and 46.3 ng $m^{-3}$ (Ni) at Sejong University, Seoul in December 2002[44]. Similarly, the mean Pb concentrations in this study are less than those measured at a residential area in the northern part of Seoul (75.3 ng $m^{-3}$) during 2005–2006[46]. This is possibly due to the fact that the phase-out effect of the dominant source processes (i.e., use of leaded gasoline) has been effective continuously throughout the years[47]. In contrast, the metal data in the urban areas in European and American countries are significantly lower than others. For example, in European countries (e.g., Sweden, Spain, Greece, and the U.K.), the metal values occurred in several-fold reduced ranges of 183–800 (Fe), 2.9–11.0 (Mn), and 3.4–40 ng $m^{-3}$ (Pb)[30,48,49,50]. Likewise, in American countries (e.g., the U.S. and Argentina), the metal values were seen in the range 178–1000 (Fe), 4.1–31 (Mn), and 2.0–330 ng $m^{-3}$ (Pb)[18,21,51]. The status of airborne metal pollution in the study areas can also be assessed in reference to some legislative guideline values. If we refer to the European guideline values of individual substances based on effects other than cancer or odor/annoyance[52], the recommended values for certain metals like Pb, Cd, and Mn correspond to 500, 5, and 150 ng $m^{-3}$ for annual averaging, respectively. Although the data obtained from some Asian sites (e.g., Hong Kong and India) exceed such criteria values, most results (including ours) seem to fall below such guideline values.

## CONCLUSIONS

The concentration data sets of 17 metals were measured from four monitoring stations located in residential areas in Seoul, Korea during a 6-month campaign period from February to July 2009. The highest mean concentrations were seen consistently from Fe at all four sites such as 2599 (JR) to 2897 ng $m^{-3}$ (YJ). On the other hand, the lowest values were seen from either Ag (0.66 [JR] to 1.11 ng $m^{-3}$ [GJ]) or Co (1.57 [GS] to 1.59 ng $m^{-3}$ [YJ]). The analysis of the metal data among the four study sites showed that the high metal levels occurred abundantly from the GJ or YJ site due to the proximity of the high traffic

**TABLE 5**
**Comparison of the PM-Bound Metal Concentration Levels Measured in Urban Areas (Commercial and Residential Area) between the Present and the Previous Studies (All Units in ng m$^{-3}$)**

| Order | Site | Study Period | Size | Fe | Zn | Cu | Ba | Mn | Pb | Ag | As | B | Cd | Co | Cr | Li | Mo | Ni | Sr | V | Ref. |
|---|---|---|---|---|---|---|---|---|---|---|---|---|---|---|---|---|---|---|---|---|---|
| *Asian countries* | | | | | | | | | | | | | | | | | | | | | |
| 1 | Four sites in Seoul, Korea | Feb.–July 2009 | TSP | 2774 | 277 | 61.8 | 78.7 | 72.7 | 67.6 | 0.89 | 6.40 | 20.0 | 2.48 | 1.54 | 12.5 | 3.81 | 2.44 | 9.59 | 12.2 | 8.57 | This study |
| 2 | Munakata, Japan | Oct. 1997–Mar. 2006 | TSP | | | | | 22.5 | | | 2.17 | | | | 4.61 | | | 3.9 | | | [41] |
| 3 | Yanagawa, Japan | Oct. 1997–Mar. 2006 | TSP | | | | | 25.4 | | | 2.08 | | | | 4.43 | | | 4.64 | | | [41] |
| 4 | Kanpur, India | Oct. 2002–Feb. 2003 | PM10 | 300–6170 | 200–1630 | | | | 70–1030 | | | | 2–43 | | 32–400 | | | 40–270 | | | [44] |
| 5 | Hok Tsui, Hong Kong, China | Nov. 2000–Feb. 2001 | PM10 | | | 15300 | | 20,620 | 62,750 | | 3620 | | | | 4970 | | | 8270 | | 3840 | [42] |
| 6 | Napporo, Japan | 1974–1996 | TSP | | 39.6 | 14.4 | | 6.47 | 17.5 | | 1.57 | | | | 1.56 | | | 2.4 | | 3.52 | [36] |
| 7 | Nonotake, Japan | 1974–1996 | TSP | | 38.3 | 8.2 | | 6.61 | 23.2 | | 2.74 | | | | 1.14 | | | 1.48 | | 2.44 | [36] |
| 8 | Sejong University, Seoul, Korea | Dec. 2002 | PM10 | 1643 | 428 | 28.2 | 38.9 | 37.6 | 200 | | | | 5.47 | 2.53 | 73.6 | | 13.5 | 46.3 | | 2.96 | [45] |
| 9 | Donga University, Busan, Korea | Dec. 2002 | PM10 | 560 | 787 | 19.2 | 11 | 45 | 128 | | | | 3.17 | 1.41 | 36.6 | | 10.6 | 22.5 | | 3.49 | [45] |
| 10 | A residential area, Seoul, Korea | 2005–2006 | PM10 | | 115.8 | | | 21.5 | 75.3 | | 2.20 | | 4.10 | | 10.6 | | | | | | [46] |
| *European countries* | | | | | | | | | | | | | | | | | | | | | |
| 11 | Stockholm, Sweden | 2009 | TSP | | 17 | 7.7 | | 5.5 | 3.4 | | 0.88 | | 0.11 | | 2.3 | | 1.6 | 2.3 | | 1.6 | [30] |
| 12 | Vila-real, Spain | 2002–2005 | PM10 | 500–600 | 86–364 | 8.0–10 | 12–120 | 8.0–11.0 | 20–40 | | 3.0–12.0 | | 0.7–2.9 | 0.6–1.1 | 4.0–7.0 | 1.0–1.6 | | 4.0–5.0 | 4.0–5.0 | 5.0–8.0 | [48] |
| 13 | L'Alcora, Spain | 2002–2005 | PM10 | 600–800 | 89–416 | 5.0–7.0 | 12–100 | 6.0–8.0 | 30–10 | | 3.0–11.0 | | 0.8–1.8 | 0.7–0.8 | 5.0–8.0 | 1.3–1.9 | | 3.0–4.0 | 4.0–6.0 | 4.0–6.0 | [48] |
| 14 | Thrakomacedones, Athens, Greece | June 2001–May 2002 | PM10 | | | 13 | | 4.4 | 25.4 | | 4.8 | | 1.9 | | 10.1 | | | 9.2 | | 3.7 | [49] |
| 15 | Edinburgh, U.K. | 1999–2000 | PM10 | 183 | 13.3 | 4.93 | | 2.9 | 14.1 | | 0.4 | | 0.34 | | 1.6 | | | 3.4 | | 1.14 | [50] |
| *American countries* | | | | | | | | | | | | | | | | | | | | | |
| 16 | Los Angeles, U.S. | 2002–2003 | TSP | | 84 | 52 | | | 14 | | | | | | 4.9 | | | 9.2 | | | [51] |
| 17 | La Plata, Argentina | 1993 | | 178–1495 | 34–658 | 7.6–163 | | 4.1–31 | 2.0–101 | | | | 0.13–1.2 | | 0.6–7.9 | | | <1.1–5.2 | | | [21] |
| 18 | Detroit, U.S. | 1982–1992 | | 300–1000 | 30–800 | | | | 40–330* | | | | 1.0–3.0 | | 8.0–14 | | | 5.0–16 | | | [18] |

*The Pb concentration in the commercial area.

density area or by outdoor incineration activities. The calculation of the enrichment factor (EF) for metals showed that the strongest enrichment tends to occur from metals like Cd, Pb, and Zn, whereas relatively small EF values are seen from such metals as Ba, Co, Cr, Li, Mn, Ni, Sr, and V at all four sites. Therefore, the metal pollution status in these areas is reflected by such source processes as vehicular activities.

A comparison of temporal patterns indicates that the largest monthly values were typically observed in spring months (e.g., April), while the lowest data tend to occur during summer months (e.g, June). The springtime enhancement in metals may be explained by the effect of AD from north China. The results of the factor analysis further revealed that the most dominant sources of metals were accounted for by resuspended soil/road dust or the combustion of fossil fuel. A comparison of the metal data between different urban areas on the globe confirms that the metal concentrations in urban areas of the Asian countries were noticeably higher than their European or American counterparts. These differences may be explained by the combination of increasing energy consumption and the limited emission control systems in Asian countries. The overall results in this study indicated that source processes of the four study sites were differentiated from each other, while the combined effects of resuspended soil dust and transport of vehicles may act as the essential components of metal pollution in urban areas.

## ACKNOWLEDGMENTS

This work was supported by a National Research Foundation of Korea (NRF) grant funded by the Ministry of Education, Science and Technology (MEST) (No. 2009-0093848).
